# More than just a task: intimate care delivery in the nursing home

**DOI:** 10.1080/17482631.2021.1943123

**Published:** 2021-06-28

**Authors:** Genevieve N. Thompson, Susan E. McClement, Sheryl Peters, Thomas F. Hack, Harvey Chochinov, Laura Funk

**Affiliations:** a College of Nursing, Rady Faculty of Health Sciences, University of Manitoba, Winnipeg, Manitoba, Canada; b Department of Psychiatry, Max Rady College of Medicine, Rady Faculty of Health Sciences, University of Manitoba, Winnipeg, Manitoba, Canada; c Department of Sociology and Criminology, University of Manitoba, Winnipeg, Manitoba, Canada

**Keywords:** Person-centred care, nursing homes, relational care, dignity, activities of daily living

## Abstract

**Purpose**: Intimate care procedures, such as bathing and toileting, are often regarded as simple, humble tasks. However, the provision of such care transforms a very private, personal activity into a social process. U*nderstanding* this complex process and the psychological impact it has on those providing and receiving care is critical in order to mitigate potential distress. The purpose of this study to examine the experience of delivering and receiving intimate personal care in the NH.

**Methods**: A focused ethnographic approach with participant observation, semi-structured interviews, focus groups and drop-in sessions, document review, and field notes. Data were analysed using constant comparative analysis.

**Results**: Quality care in this context is predicated on the care provider recognition of the emotional impact of care delivery on the care recipient. Our analysis identified that the overarching theme, of providing quality person-centred intimate care, requires creating and maintaining a relational space that promotes integrity.

**Conclusions**: The provision of intimate personal care consists of a complex interplay at the level of resident/care provider interaction (micro level); health care organization (meso level); and policy (macro level). Each of these levels interacts with and influences the other two. The components identified in our model may provide the basis from which to further examine resident experiences of quality intimate personal care.

## Introduction

Discussions around intimate care dependencies, those activities of daily living such as bathing, toileting, dressing and feeding, evoke strong emotions in individuals who are recipients of such care (Yeung et al., 2019). No one wants to be dependent on others for these basic human necessities; yet the majority of those residing in nursing homes (NHs) require assistance with one or more of these activities (Remillard et al., 2019). Being dependent is one of the greatest fears of older people, and results in feelings of being a burden to others and psychological distress (Abad-Corpa et al., 2012).

Intimate care procedures are often regarded as simple, humble tasks, and are conferred less prestige than procedures requiring technical nursing skills (Picco et al., 2010). In both Canada and the USA, an estimated 80% to 95% of direct resident care, including intimate personal care in NHs is provided by nursing assistants (NAs) (Berta et al., 2013; Hewko et al., 2015). As frontline caregivers, NAs generally have some secondary education which focuses primarily on the basic physical care of the resident, with minimal attention directed towards psychosocial, spiritual, or person-centred care (Barken & Armstrong, 2018). Too often, in the NH setting, intimate personal care becomes task-based and occurs in environments that prioritize efficiencies over emotional care (Rodriquez, 2011).

The provision of intimate personal care transforms a very private, personal activity into a social process shaped by a complex interplay of factors related to health care provider (HCP) attitudes and behaviours, resident characteristics, the physical care environment, and organizational cultures (Sacco-Peterson & Borell, 2004). An empirical understanding of this complex process and the psychological impact it has on those providing and receiving intimate personal care is critical in order to mitigate potential distress and suffering. However, there is a paucity of research examining the complex factors that shape NH resident and HCPs’ experiences with the intimate care provision (Baillie, 2009; Twigg et al., 2011). To address the limitations of existing research in this area, this study aimed to examine the experience of delivering and receiving intimate personal care in the NH. More specifically, we wished to understand what constitutes quality person-centred intimate care within the context of NH care.

## Methods

To gain a deeper understanding of how NH residents and HCPs experience intimate care, a focused ethnographic approach was chosen, based on Roper and Shapira’s (2000) framework. Data collection consisted of participant observations and semi-structured interviews with both residents and HCPs, focus groups and drop-in sessions with HCPs, document (e.g., mission, vision, and value statements of the facilities), reviews and field notes, all conducted by research assistants (RA) hired for the study between March 2015 and June 2017. Data were collected at the first site before starting at the second. This study received ethical approval from the University of Manitoba Education/Nursing Ethics Board.

### Setting and participants

The study was conducted at two NH sites in a large urban city in Central Canada, chosen for their mix of residents with varying degrees of care dependency. Differences in environmental factors, such as facility layout, size and structure between the two sites allowed for an exploration of the impact of the physical and organizational environments on the experiences of receipt and delivery of intimate care. Study sites were also selected based on their willingness to grant the PI entrée and staff receptivity. Both sites are privately managed by the same NH company and receive partial public funding. Each site houses approximately 175–200 residents.

At each site, the first author and main study RA conducted a presentation regarding the study at a staff meeting. Handouts with study information were left for those not in attendance. Inclusion criteria for HCP participation consisted of 1) providing direct care to residents; and willingness to: 2) provide informed consent; 3) have research staff “follow them during their shift”; and 4) participate in informal and formal interviews.

Administrative decision-makers such as nurse managers, directors of nursing, chief executive officers, and programme managers were also invited to participate in key informant interviews. Inclusion criteria for administrative staff consisted of 1) having administrative responsibilities for direct patient care activities; and willingness to 2) provide informed consent and 3) participate in a semi-structured interview.

For residents, the process for participation involved two potential options: to be part of the observational portion of the study and/or participate in a semi-structured interview. Inclusion criteria for residents consisted of 1) having an intimate care dependence; 2) being 18 years of age or older; 3) able to understand English; and 4) cognitively competent to participate (based on clinical consensus).

As many residents of NHs have some degree of cognitive impairment, the project obtained clinical consensus from the nursing manager on whether the resident could consent on their own. If consent from the family was required, NH staff contacted family members, provided a brief description of the research, and asked them if they would agree to have their contact information provided to the main study RA. This RA then approached the families, described the research in detail, answered any questions and gained written consent from a family member. Verbal consent was obtained from all cognitively intact resident participants, and verbal assent obtained from residents who were cognitively impaired, but whose families had consented on their behalf.

Family members of residents were also invited to participate through: letters sent from the NH along with regular NH communications; email and telephone contact from staff; or being approached by the main study RA while visiting the facility. Inclusion criteria for participation included: 1) knowing details regarding the resident’s day-to-day care in the facility; and 2) willingness to provide consent and participate in an interview.

### Procedure

In keeping with a focused ethnographic approach (Roper & Shapira, 2000), the study started with both the first author and the main study RA, conducting participant observation at each site. The purpose of this initial observation was to gain a coherent sense of the whole, that is, to gain an understanding of the ebb and flow of the work and a sense of the general layout of each facility. Documents were collected and reviewed to better understand the information provided to staff relevant to intimate care and person-centred care and included the mission, vision, and value statements of the facilities, along with creating sketches of the physical layout of the NHs.

Once the initial broad observation was completed, participant observation of the care environment and focused participant observation of intimate care delivery was conducted with care providers and residents. Observations of the care environment took place at various times of day and night over the period of the study, and included: viewing the physical environment, observing and participating in interactions in public areas, and observing nursing stations to better understand the impact of the physical environment and organizational characteristics on the delivery and receipt of care.

Focused participant observation of intimate care delivery took place in resident rooms. This was a negotiated process. A staff member appointed by the Nursing Manager of each site (or their designate) reviewed the charts of residents to identify those who satisfied the inclusion criteria. The staff member approached eligible residents and sought permission to release their name to the main study RA. This RA approached the potential respondents, gave a brief description of the study and the observation requested, and determined if they met study criteria. NAs and nurses assigned to care for eligible residents were approached, given a brief description of the study, and asked permission to observe their caregiving. When a resident/HCP dyad gave permission to participate in the observation, the main study RA joined the dyad in the resident’s room and asked detailed permission of the resident, giving them a number of options to have their care receipt observed (for example, to allow observation of all care, to allow observation of some care acts and not others, to allow watching or just listening). Most often, care such as toileting and bathing was negotiated as being able to stand outside the door and listen; care such as dressing, feeding and transfers was fully observed. Observational data were recorded as hand-written field notes, focusing on the interaction between the resident, care provider and the organizational system.

After the observation, when appropriate, the resident was asked if they would also like to participate in a research interview at a time convenient for them. Other residents, for whom observations were not conducted, were recruited by a staff member appointed by the Nursing Manager of each site (or their designate) who reviewed the charts of residents to identify those who satisfied the inclusion criteria. Staff members approached eligible residents and sought permission to release their name to the main study RA. This RA approached the potential respondents, gave a brief description of the study, and determined if they met study criteria. Once permission was given, the main study RA set an interview time.

For HCPs, recruitment for the semi-structured interviews was achieved through a variety of means including posters and an email sent on the behalf of the researchers by the administrative managers. HCP interviews were conducted either at the end of their shifts in a room at the NH or on a day off in their home. Interviews focused on HCP/resident relationships, particularly around intimate care needs; teamwork between staff; HCP/management relations; and how the physical environment interacts with living, working and caregiving activities.

Due to the low numbers of HCPs participating in the semi-structured interviews, several focus groups were run. Three focus groups, one with NAs and two with nurses, were conducted with the researcher and main study RA at one of the study sites. Additionally, three drop-in research sessions were conducted by the main study RA and an additional RA, one on each shift over the course of three days. HCPs came to a large room during their breaks and received seven 4”x6” blank post-it notes to provide responses to seven research questions (see Table I). The research questions were written on large posters set on tables. HCPs were encouraged to circulate around the tables, prepare written responses on the 4” x 6” post-it notes and adhere them to the corresponding poster question. These two RAs were available to provide direction and support to respondents as needed throughout the event.

**Table I.** Research questions used in focus groups and drop-in sessions

1) What makes a good nursing aide?
2) What makes a good nurse?
3) What kinds of things do you do that makes a resident feel cared for?
4) How do you manage your feelings when you see or smell things in the course of providing care that some people might consider smelly or disgusting?
5) What frustrates you about your job?
6) Often when providing care, there will be several residents who require your attention all at the same time. How do you decide whom to respond to first?
7) What would be on your “wish list” if you could change anything to enhance the care of residents at (this NH) or to enhance the workplace?

Table II provides a detailed account of the frequency of the various data collection methods employed in this study.

**Table II.** Frequency of data collection methods

Informal observations	600 hours
Formal observations	9 hours
Number of different residents formally observed	11
NA interview duration time range	20–240 minutes
Nurse interview duration time range	60–90 minutes
Resident interview duration time range	30–90 minutes
Family interview duration time range	45–150 minutes
Focus group duration	90 minutes
Drop-in research event duration	12 hours

### Data analysis

The purpose of ethnographic analysis is to organize the data and then to make sense of what we have learned during the research process by categorizing the data into meaningful pieces, and then examining those segments for patterns that explain the phenomena of interest (Cruz & Higginbottom, 2013). Using the strategies for ethnographic analyses outlined by Roper and Shapira (2000), the inductive analysis of data, using constant comparative content analysis (Coffey & Atkinson, 1996) involved coding field notes and interviews, sorting to identify patterns, generalizing constructs and theories, and memos to note personal reflections and insights. Transcripts were read in their entirety in order to gain a sense of the whole and to identify keywords, phrases, or emerging themes to form codes (Speziale & Carpenter, 2007). Categories were developed by clustering coded data into meaningful groups and the basic properties of the categories defined, relationships between categories identified, and categories were compared to ensure they were mutually exclusive. Descriptions of each theme or pattern and their relationships with each other were written in order to provide a coherent picture of person-centred intimate care provision and the barriers and facilitators to achieving quality care in this regard. Data coding was conducted by Thompson and McClement, along with the two RAs hired for the study using discussion to reach consensus. The rest of the research team reviewed the coding scheme for coherence and clarity, and served in the capacity of objective persons to check possible biases and assumptions that may have manifested in the coding scheme.

## Results

There were a number of ways to participate in the study as described in Table III. On average, resident participants were 83 years of age and female. Of the family member participants, 58% were female, with an average age of 50. Most described visiting their family members in the facility every few days (50%), once a week (33%) or daily (16%). Table IV describes the characteristics of HCPs and administrators who participated.

**Table III.** Details of participation in data collection

	NAs	Nurses	Administrative Decision-Makers	Residents	Family Members
Participants in formal observations	7	1	N/A	11	N/A
Number of Interviews	13	5	5	18	12
Participants in focus groups	6	8	N/A	N/A	N/A
Participants in drop-in Research Event	55	16	N/A	N/A	N/A

Some of the NA and Nurse interview participants also participated in the drop-in event.

**Table IV.** Staff participant demographics

	Total (N)	Gender	Cultural Identity	Average Age (Yrs) (range)	Average Years in Health Care (range)	Average Years in Current Position (range)
NAs	74	Female:63 Male: 11	59 Filipino 3 Indian 4 African 1 Caribbean 1 Black 6 Caucasian	48 (28–60)	18 (2–40)	15 (<1–34)
Nurses	29 RN = 17 LPN = 12	Female:23 Male: 6	21 Filipino 1 Indian 1 Filipino/Chinese 1 Chinese 1 Black 1 Caucasian 1 Multiple 2 Undeclared	42 (24–67)	15 (1.5–37)	9 (1.5–25)
Administrative Decision-Makers	5 RN = 3 Other = 2	Female: 3 Male: 2	3 Filipino 2 Caucasian	37 (32–45)	19 (11–24)	10 (6–14)

### Relational space

**Figure 1.**
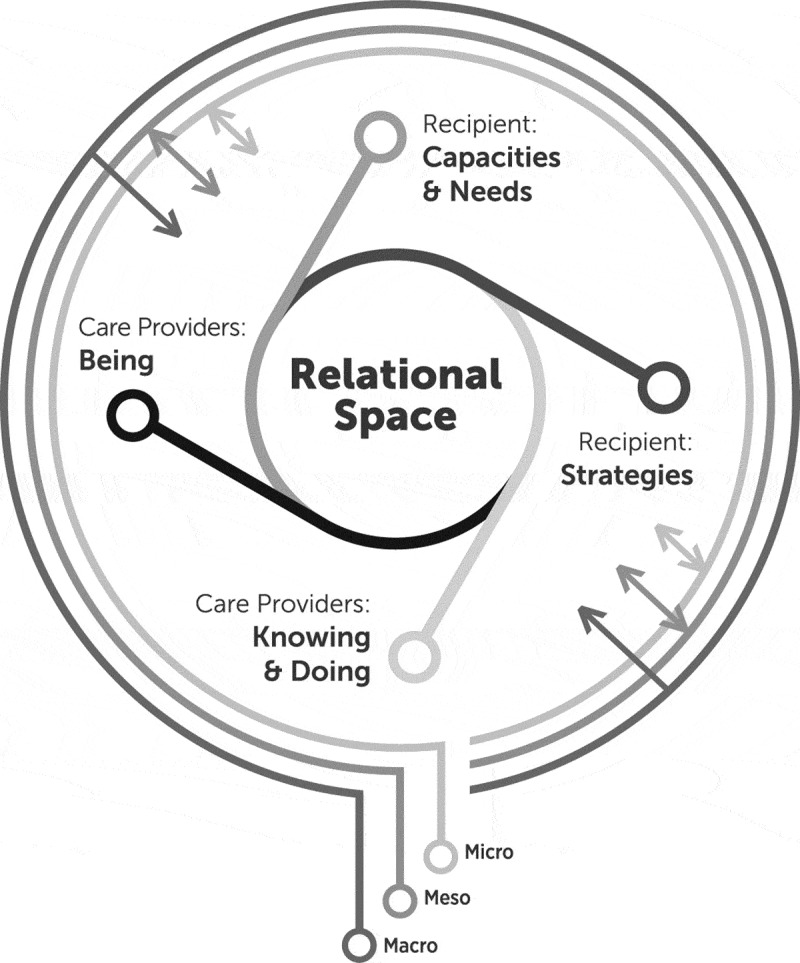
Creating a relational space to promote integrity in the provision of intimate care

Our analysis of the data identified that the overarching theme, of providing quality person-centred intimate care, requires *creating and maintaining a relational space that promotes integrity* (see Figure 1). Quality care in this context is predicated on the HCP recognition of the emotional impact of care delivery on the care recipient. In order to create this relational space, HCPs respond through their *being—*that which is innate in the care provider; and by *doing—*operationalizing the knowledge and understanding they bring to care provision. The *needs and capacities* of the care recipient affect the quality of interaction between them and the HCP. The care recipient may engage in intentional actions (*strategies*) to help ensure their physical and psychosocial well-being is maintained; though this role may be taken on by family when communicative abilities in the resident have declined. Finally, the aggregate of dynamic physical, social, and cultural conditions and internal and external factors (*micro, meso and macro environment*) can influence individuals and/or the care setting to shape the quality of intimate care. Each of these sub-themes is further described below, supported with data exemplars.

### Care providers: being

Care providers, and NAs in particular, before they even start their shifts, have a set of intentions that are part of who and what they are about and what they want to achieve. These intentions seem to be the foundation from which they act. This is “*being*”; it’s about who they are. At its core, HCP *being* encompasses the trained and embodied capacities, tendencies, propensities and predispositions of the HCP that guide their thoughts, feelings, and actions. These learned and innate behaviours are manifest through who the HCP is as a person and the values they espouse. These behaviours have been shaped through their experiences, both in their personal lives and through their education, training and working life.

Excellence in intimate care provision required HCPs to be personally invested in their work. These providers spoke with passion about their role, their love of caring for residents and the importance of being committed to their job. As one participant said: *You have to have the passion to do your job. Because if you have the passion to do your job, any challenge you have you will be able to look at it in a positive way. ID 14 (82–83)*

Many of these embodied capacities are consistent with our understandings of compassion; that is, HCPs espoused the qualities of emotional presence, being respectful and sensitive, humbleness and humility, showing love to the resident and their colleagues, thoughtfulness and kindness. This disposition fostered HCPs to be patient, tolerant, thoughtful, and non-reactionary when providing care. *“Always think before you act. And before you say a word try to think first and weigh the situation”. ID 14 (654)*

HCPs who used their *being* to create relational space had a collective vision—they were not “I” driven, and their work was not about them. Rather, they focused on the needs of the resident and in helping out their colleagues. In this way, they saw the sharing of roles as important to achieving excellence in care. *“ … when it comes to working together well you can’t turn around and say I’m not doing this job and it is left … ” ID 22 (588)*

### Care providers: knowing & doing

Out of the *“being”*, the “*doing*” flows. Excellence in intimate care requires that HCPs know what outcome they are looking for and work steadfastly towards achieving it. The process of achieving optimal care outcomes requires both intention and attention.

Intentional means be more conscious of what or when you approach a resident. It is not that a resident is another room number so you have to introduce yourself, you have to ask them their preferences, you have to anticipate their needs. ID 27 (293-301)

This intention and attention is what sets excellence in care apart from rote task and “doing to”. There is a negotiated stance that the HCPs engage in to create relational space. It involves mutual respect and the idea that both resident and HCP has something important to offer in the relationship. As much as possible, HCPs try to elicit what the preferences and needs are of the care recipient whilst at the same time balancing the tension of needing to get their work done.

Some of them don’t want to cooperate with you so I have to find a way to encourage them to cooperate. I use my imagination. And I use my observations. I try to know each single resident, what their weaknesses are and I also try to remember their routines … So you negotiate with the residents to make your work manageable because you have to remember that you have different responsibilities over your eight-hour shift so you have to find a way around it to make yourself useful and to be able to accomplish your responsibilities as well. ID 14 (21-80)

There are two parts to using intention when caring for a resident with attention—getting to know the resident initially and engaging in day-to-day attentiveness when providing care. HCPs and NAs in particular, try to foster an independence in the resident, but they also make a moment-to-moment assessments of the residents’ abilities. They assess the situation by “tuning in” day-to-day with resident ability/capacity (assessment piece) and they modify/adjust what they do with and for the resident to what is needed in the moment based on that assessment. HCPs are neither “stuck in their ways” nor do they use a “one size fits all” approach to care. They have to be focused on the resident—all “eyes and ears” in thinking about resident needs in the moment.

Within this knowing and driven by their being, HCPs engage in specific intentional actions or approaches (doing) when providing intimate care delivery. *Responding to* was a significant action and consisted of the ability to reassure, normalize, explain, downplay, use humour and coaching when providing care. The actions were used by NAs as they strove to minimize the emotional impact and possible distress that could be experienced when receiving intimate care.

Give them privacy … we tell them ‘It is okay. That is why we are here to help you. You don’t have to worry. Things happen. Maybe this time it happened, maybe tomorrow it won’t happen. We are here to help you. We are trained for it. So you don’t have to be shy or you don’t have to be embarrassed.’ So they realize that it is okay. And if they are incontinent and in the later stages they get incontinent they start crying and say “Oh, I’ve never peed in my pants before. All of my clothes are wet.” It is okay, don’t worry. We’ll fix it. It happens. ID 10 (259-265)

An important aspect of knowing and doing is the recognition by the HCP that they are the instrument of care. They recognize their own needs and used specific strategies such as being in the moment, managing their own emotions, not taking the reactions of residents personally and collecting or centring themselves.

I always say to myself if I am losing my patience or whatever I have to heal myself. I have to take a deep breath sometimes and if it is a very challenging one, I will say “Do you mind excusing me for a minute?” And then I will go in the hallway and then I will take a deep breath and then ahh. ID 14 (167-168)

### Recipient: capacities & needs

Residents have unique capacities and needs, which are either expressed or perceived. Resident capacities and needs can be health related, such as physical, sensory and cognitive factors, and psychosocial factors. The nature of these needs and HCP responses to them has the potential to bolster or erode the creation of relational space and in turn, the integrity of the resident. For example, hearing and vision loss can greatly influence the resident’s ability to interact in a meaningful way with HCPs. One resident shared that because of her vision loss, she identifies when different staff are on shift by their voice but can’t read nametags. Unless they tell her who they are and are up close, she is unable to know who they are. What was upsetting to her is that she is chastised by the staff for not knowing or remembering who each staff member entering her room is (ID 28—observation notes).

Residents negotiate a host of psychosocial factors when entering into and living in a NH. The responses of HCPs shape the ways residents experience these challenges. For example, some residents fear was predominate in their first weeks in a NH. One resident recalled, *“When I came here, I was scared of everything. I was scared to let myself fall asleep because I thought that I would never wake up, you know.”*

Many residents experience a sense of loss, embarrassment, and feeling like a burden when they cannot do things they used to do for themselves. Incontinence is a frequently cited source of psychosocial distress. Residents spoke of embarrassment for having incontinence and feeling like a burden for needing care. When HCPs were not responsive to the emotional significance of incontinence, residents reported feeling dismissed when being told to wait by busy staff, or a lack of dignity—”go in your brief”, or feeling pressured to hurry when they were being toileted because staff are rushing. When HCPs are aware of and respond with compassion to these feelings, they reassure and normalize intimate care for residents.

### Recipient: strategies

Residents have agency and are often active in the process of negotiating care to ensure their physical and psychosocial well-being is maintained.

Like I mean pay attention. That is the thing. Like if I am telling you something, listen to what I am telling you. You don’t know how I feel because you possibly cannot. But listen. ID 28 (162-164)

Sometimes this is done verbally and in other instances it is accomplished through their behaviours. *“In people that are cognitively impaired … once they are in pain, they cannot express it and they will not assist you. They will tense. But if you are gentle with them they can sense just by touching them.” ID14 (1016–1018)*. In those instances of behavioural expression, HCPs engage in the process of *knowing and doing* to decipher residents’ needs. *“I want to understand the residents, why they are acting the way that they are and what is the trigger to their behavior because those are things that you have to observe.” ID 14 (136–138)*

When verbally able to, residents will often direct staff regarding their care provision, for example, articulating when they would like to get out of bed, what tasks they prefer to do themselves, or how they would like care tasks done. Residents valued feeling heard in this regard: *“Well, like they ask me ‘Are you okay? Is that too high?’ If I say yes, then they lower my head or my legs or whatever.” ID 28 (71–72)*

In residents with higher levels of cognitive capacity, residents spoke of the importance of engaging in a reciprocal relationship with staff, ascribing to the belief that by giving respect to the HCP, they would be respected in return. More cognitively well residents also noted the importance of advocating for others who may not be able to speak up for themselves. However, the data did show the use of passive strategies residents used in care negotiation such as not complaining or being resigned to whatever care was provided.

I mean the way that they put me in here this morning, I will stay like that until they put me in another position because I can’t move those legs. They are totally useless. So I mean it is difficult but I am alive. ID 28 (28-30)

Several of the families shared with the RA informally that their family members who are residents did not complain much, even when there were problems with care. The families had to advocate because the resident’s reluctance or inability due to their condition to speaking up to the care providers.

### Environment: micro, meso, macro

When thinking of person-centred care, we often think of the one-to-one care encounter between HCP and care recipient. At that micro level, it is important to remember that the environment also influences the creation of a relational space, and delivery of excellence in care is shaped by the physical layout of the facility, the availability of supplies (e.g., linens and gloves), the amount of paperwork required to complete, resident acuity, and workload. For example, the facility that was quite vertical with many small floors had the advantage of being able to group residents with similar care needs (e.g., levels of dementia and behaviours). Less sound travelled between the floors to residents who were cognitively competent from residents who, because of their dementia, had loud or repetitive utterances. Therefore, it was quieter than then NH with more horizontal layout that had two large floors. However, the more vertical facility had tiny elevators which took so long to move people between the floors that it impacted the activities of those living, visiting and working there. The facility with the two larger floors had few mobility issues and more capacity for activities and work in a timely manner.

An example of how workload can affect the disposition of the staff and translate to the residents is found in the following quote from a HCA:

Sometimes we don’t realize we are loud and the way that we say good morning it is time to get up. … Some staff, because of all the workload that you have for the day, your adrenaline is pumping and you are over assertive. ID 14 (971-983)

The environment at the facility level (or meso level) also exerts a strong influence on relational care. The most significant driver in this area is nursing home leadership. Management played a key role in setting the tone for their administrative and front-line staff in regards to the philosophical approach to care practices. In creating relational space, practices that fostered a relationship centred culture included meeting regularly with staff, listening to employees, being genuine and respectful, mentoring staff, showing appreciation, and following up after having implemented change. Inviting feedback from all parties and seeking collective solutions tended to result in a more person-centred environment since when staff feel that they can positively influence the conditions of care, they have more incentive to go beyond instrumental and technically proficient care and tend to go “the extra mile” for residents. An example provided by both a manager and HCA was the story about the resident and her suitcase. One of the facilities brought in a pilot program to encourage person-centred care. Staff were encouraged to come up with small initiatives and a small fund was available, though many of the initiatives did not cost any money. One resident with dementia was repeatedly looking for a black suitcase, which had not actually come with her to the facility, but she would become increasingly agitated when it could not be found. This issue caused her great distress and required a great deal of attention from staff. The initiative empowered the health care aides to come up with a solution, so they purchased an inexpensive black suitcase for the resident, so she would be soothed.

Finally, at the broadest level, the system (or macro) itself exerts influence on creating relational space. Nursing homes are heavily regulated environments that sometimes preclude adoption of more person-centred or relational practices. Staffing ratios, which have remained unchanged for decades despite increased acuity, along with stagnant government funding to NHs impact care provision. For example:

If we are understaffed it (giving person-centered care) is so difficult. It (being understaffed) wears out the staff as well. It is a vicious cycle. Residents complain, staff don’t feel good about it, and then they sometimes call in sick because of that. ID 27 (536-538)

## Discussion and implications

Providing intimate personal care “is about hands-on work which invades accepted personal and social space” (Carnaby & Cambridge, 2002, p. 126). Our findings identified that the provision of quality person-centred intimate care occurs in a relational space that maintains resident integrity. Quality care is thus comprised not only of *what* procedures and interventions are delivered but also *the way* in which they are delivered (Ferri et al., 2015; Šaňáková & Čáp, 2019). The provision of quality intimate personal care consists of a complex interplay at the level of resident/HCP interaction (micro level); health care organization (meso level); and policy (macro level). Each of these levels interacts with and influences the other two. We appreciate that the delineation between the levels is not always clear. For example, lack of HCP requisite knowledge and training to be able to provide care would be characterized as a micro-level problem because it affects residents. Knowledge and training deficiencies can be considered a meso-level issue as it is the duty of the NH to ensure that employees have the training they need to care for residents. HCP training can also be a macro-level issue as policies may dictate the content of training programmes and the requirement for ongoing education for employees. Thinking about the provision of quality intimate personal care in terms of these interrelated levels offers an important vantage point from which to consider how to drive optimal care outcomes forward.

The maintenance of integrity as an important precondition for quality care in long-term care settings has been previously noted in the literature (Randers & Mattiasson, 2000; Teeri et al., 2007). Our data demonstrated that this outcome does not occur by happenstance; rather, HCPs were intentional about their goals in interacting with residents. Far from executing tasks by rote using a one size fits all approach, HCPs were actively engaged in getting to know the resident and understand their needs and preferences. They collected and used information about the resident’s unique needs and preferences to provide individualized care. Knowing the patient has been described in the literature as a central phenomenon used by nurses in decision-making to provide good and safe quality individualized care (Johansson & Mårtensson, 2019).

Consistent with findings of Vassbø et al.’s (2019) study of the meaning of working in NHs, participants in our study emphasized the importance of recognizing resident preferences, and the need for flexibility and creativity when providing care. Such an approach to care is integral in reinforcing autonomy, choice and control, and contributes to resident well-being (Edvardsson, 2015). The tenor of care provided was characterized by HCP attributes of respect and kindness. Being respectful and responsive to individual preferences and needs are characterized in the literature as central features of good quality care, supportive of patient dignity and hallmarks of therapeutic relationships in the caring professions (Kornhaber et al., 2016; Thompson et al., 2011).

The being, knowing, and doing identified in our study closely parallels Steinke’s (2016) observation that, “the science of nursing pertains to what is done, and how; the art of nursing focuses on who is doing it and why” (p. 34). These key themes are also resonant with the dimensions of Sinclair et al. (2016) empirically derived model of compassion in clinical practice. First, our theme of “being”—the way HCPs behave because of who they are as a person, is consistent with what is described in the model as HCPs’ embodied virtues, or noble qualities such as genuineness, understanding, kindness and acceptance as being dispositional, and independent of patient behaviour. Secondly, both studies identified the construct of a relational space as the context in which care, characterized by highly engaged HCPs seeking to know the patient as person, understand and respond to unique needs, identifying and responding to needs. The positive outcomes in our study resonate with the work of Sinclair et al. (2016) and included residents feeling heard and respected, and the preservation of psychological integrity.

Our findings surfaced the complex and intricate interaction between resident and HCP that affects the experience of receiving intimate personal care. Care provision in NHs is described as stressful, and the literature is replete with references to lack of staff, high workload, resident aggression and employee burnout (Gaudenz et al., 2019; Yeatts et al., 2018). While real, a predominant focus on the burdens and challenges of providing care fails to capture the dynamics between the resident and HCP. The important context of the relationship and the perspectives and interpretations that residents and HCPs each bring in creating the interaction is negated (Tallman, 2015). Our findings demonstrated that in deploying certain strategies aimed at shaping the interactions they had with HCPs, residents were very much co-participants in their care. Some resident participants in this study used the strategy of providing direct and explicit verbal communication to ensure HCPs delivered care in accordance with resident wishes. Other residents focused on forging respectful relationships with their caregivers, while others acquiesced to whatever care was provided. These latter two strategies are akin to the relationship-oriented strategies identified in Evans et al.’s (2004) phenomenological exploration of resident coping strategies in the NH in response to dietary services. Residents in that study worked to forge friendly relationships with staff as a way of trying to get them to understand care needs and preferences. When overtures of friendship were reciprocated, residents complimented staff on their caring and efficiency. Seeking supportive relationships with staff and “getting along” were also strategies identified in the a qualitative study of strategies used by older people that contribute to adaptation to NH life conducted by Brandburg et al. (2013).

While many of the findings in this study are consistent with previous literature, our model present a unique opportunity for future research to explore quality intimate care from multiple vantage points. Future studies could be developed to target unique interventions aimed at fostering relational space in the context of nursing homes and the optimal conditions to achieve it. It will be important to examine outcomes both for the resident and care provider when relational space to promote integrity is achieved as there is the potential to significantly improve the quality of care for the resident and the quality of work life for staff.

### Limitations

Although efforts were made to maximize diversity, our participants may not be representative of the residents, family caregivers and HCPs in other jurisdictions. As focused ethnographic studies are contextual by nature, findings are not generalizable; however, similar settings could make use of the results (Higginbottom et al., 2013). To maximize transferability of the findings to similar contexts, rich descriptions have been provided (Lincoln & Guba, 1985). Finally, the data collection period in each NH may also have been insufficient, and new scenarios may have occurred if it had been extended. Despite these potential limitations affecting the trustworthiness of the findings, this study sheds an important light on the nature of intimate care provision in the NH context.

## Conclusion

This study has provided an in-depth account of the dynamics of intimate personal care by considering the perspective of the HCP and the experiences of the care recipient, all within the context of a complex care environment. The components identified in our model may provide the basis from which to further examine resident experiences of quality intimate personal care and inform the development of a measure to empirically capture those experiences. Ultimately, however, any interventions aimed at enhancing the quality of care provided must take into account the complex and reciprocal interaction of behaviour and environment.

## References

[cit0001] Abad-Corpa, E., Gonzalez-Gil, T., Martínez-Hernández, A., Barderas-Manchado, A. M., De la Cuesta-Benjumea, C., Monistrol-Ruano, O., & Mahtani-Chugani, V. (2012). Caring to achieve the maximum independence possible: A synthesis of qualitative evidence on older adults’ adaptation to dependency. *Journal of Clinical Nursing*, 21(21–22), 3153–10. 10.1111/j.1365-2702.2012.04207.x 23083390

[cit0002] Baillie, L. (2009). Patient dignity in an acute hospital setting: A case study. *International Journal of Nursing Studies*, 46(1), 23–36. 10.1016/j.ijnurstu.2008.08.003 18790477

[cit0003] Barken, R., & Armstrong, P. (2018). Skills of workers in long-term residential care: Exploring complexities, challenges, and opportunities. *Ageing International*, 43(1), 110–122. 10.1007/s12126-017-9285-7

[cit0004] Berta, W., Laporte, A., Deber, R., Baumann, A., & Gamble, B. (2013). The evolving role of health care aides in the long-term care and home and community care sectors in Canada. *Human Resources for Health*, 11(1), 25 10.1186/1478-4491-11-25 PMC3723545 23768158

[cit0005] Brandburg, G. L., Symes, L., Mastel-Smith, B., Hersch, G., & Walsh, T. (2013). Resident strategies for making a life in a nursing home: A qualitative study. *Journal of Advanced Nursing*, 69(4), 862–874. 10.1111/j.1365-2648.2012.06075.x 22812933

[cit0006] Carnaby, S., & Cambridge, P. (2002). Getting personal: An exploratory study of intimate and personal care provision for people with profound and multiple intellectual disabilities. *Journal of Intellectual Disability Research*, 46(Pt 2), 120–132. 10.1046/j.1365-2788.2002.00358.x 11869382

[cit0007] Coffey, A., & Atkinson, P. (1996). *Making sense of qualitative data: Complementary research strategies*. Sage.

[cit0008] Cruz, E. V., & Higginbottom, G. (2013). The use of focused ethnography in nursing research. *Nurse Researcher*, 20(4), 36–43. 10.7748/nr2013.03.20.4.36.e305 23520711

[cit0009] Edvardsson, D. (2015). Notes on person-centred care: What it is and what it is not. *Nordic Journal of Nursing Research*, 350(2), 65–66. 10.1136/bmj.h160

[cit0010] Evans, B. C., Crogan, N. L., & Shultz, J. A. (2004). Resident coping strategies in the nursing home: An indicator of the need for dietary services change. *Applied Nursing Research*, 17(2), 109–115. 10.1016/j.apnr.2004.02.003 15154123

[cit0011] Ferri, P., Muzzalupo, J., & Lorenzo, R. D. (2015). Patients’ perception of dignity in an Italian general hospital: A cross-sectional analysis. *BMC Health Services Research*, 15(1) 41. 10.1186/s12913-015-0704-8 PMC4312597 25627836

[cit0012] Gaudenz, C., De Geest, S., Schwendimann, R., & Zúñiga, F. (2019). Factors associated with care workers’ intention to leave employment in nursing homes: A secondary data analysis of the swiss nursing homes human resources project. *Journal of Applied Gerontology*, 38(11), 1537–1563. 10.1177/0733464817721111 28715925

[cit0013] Hewko, S. J., Cooper, S. L., Huynh, H., Spiwek, T. L., Carleton, H. L., Reid, S., & Cummings, G. G. (2015). Invisible no more: A scoping review of the health care aide workforce literature. *BMC Nursing*, 14(1), 38. 10.1186/s12912-015-0090-x 26203297 PMC4511030

[cit0014] Higginbottom, G., Pillay, J., & Boadu, J. Y. (2013). Guidance on performing focused ethnographies with an emphasis on healthcare research. *The Qualitative Report*, 18(9), 1–16. 10.46743/2160-3715/2013.1550

[cit0015] Johansson, B., & Mårtensson, L. B. (2019). Ways of strategies to knowing the patient described by nursing students. *Nurse Education in Practice*, 38, 120–125. 10.1016/j.nepr.2019.06.003 31260879

[cit0016] Kornhaber, R., Walsh, K., Duff, J., & Walker, K. (2016). Enhancing adult therapeutic interpersonal relationships in the acute health care setting: An integrative review. *Journal of Multidisciplinary Healthcare*, 9, 537–546. 10.2147/JMDH.S116957 27789958 PMC5072574

[cit0017] Lincoln, Y. S., & Guba, E. G. (1985). *Naturalistic inquiry*. Sage Publications.

[cit0018] Picco, E., Santoro, R., & Garrino, L. (2010). Dealing with the patient’s body in nursing: Nurses’ ambiguous experience in clinical practice. *Nursing Inquiry*, 17(1), 39–46. 10.1111/j.1440-1800.2009.00481.x; 20137029

[cit0019] Randers, I., & Mattiasson, A. C. (2000). The experiences of elderly people in geriatric care with special reference to integrity. *Nursing Ethics*, 7(6), 503–519. 10.1177/096973300000700606 11221392

[cit0020] Remillard, E. T., Fausset, C. B., Fain, W. B., & Bowers, B. J. (2019). Aging with long-term mobility impairment: Maintaining activities of daily living via selection, optimization, and compensation. *The Gerontologist*, 59(3), 559–569. 10.1093/geront/gnx186 29165560

[cit0021] Rodriquez, J. (2011). “It’s a dignity thing”: Nursing home care workers’ use of emotions. *Sociological Forum*, 26(2), 265–286. 10.1111/j.1573-7861.2011.01240.x 21743774 PMC3131004

[cit0022] Roper, J. M., & Shapira, J. (2000). *Ethnography in nursing research*. Sage Publications, Inc.

[cit0023] Sacco-Peterson, M., & Borell, L. (2004). Struggles for autonomy in self-care: The impact of the physical and socio-cultural environment in a long-term care setting. *Scandinavian Journal of Caring Sciences*, 18(4), 376–386. 10.1111/j.1471-6712.2004.00292.x 15598245

[cit0024] Šaňáková, Š., & Čáp, J. (2019). Dignity from the nurses’ and older patients’ perspective: A qualitative literature review. *Nursing Ethics*, 26(5), 1292–1309. 10.1177/0969733017747960 29471725

[cit0025] Sinclair, S., McClement, S., Raffin-Bouchal, S., Hack, T. F., Hagen, N. A., McConnell, S., & Chochinov, H. M. (2016). Compassion in health care: An empirical model. *Journal of Pain and Symptom Management*, 51(2), 193–203. 10.1016/j.jpainsymman.2015.10.009 26514716

[cit0026] Speziale, H. J. S., & Carpenter, D. R. (2007). *Qualitative research in nursing: Advancing the humanistic imperative* (4th ed.). Lippincott Williams & Wilkins.

[cit0027] Steinke, C. (2016). Elemental nursing: Knowing, doing, being. *Canadian Nurse*, 112(2), 34. https://www.canadian-nurse.com/

[cit0028] Tallman, B. (2015). Personhood and intersubjectivity. *Perspectives: The Journal of the Gerontological Nursing Association*, 38(1), 19–21. https://cgna.net/journal.

[cit0029] Teeri, S., Välimäki, M., Katajisto, J., & Leino-Kilpi, H. (2007). Nurses perceptions of older patients integrity in long-term institutions. *Scandinavian Journal of Caring Sciences*, 21(4), 490–499. 10.1111/j.1471-6712.2007.00499.x 18036012

[cit0030] Thompson, G. N., McClement, S. E., & Chochinov, H. M. (2011). How respect and kindness are experienced at the end of life by nursing home residents. *Canadian Journal of Nursing Research*, 43(3), 96–118. https://cjnr.archive.mcgill.ca/issue/view/223. 21977728

[cit0031] Twigg, J., Wolkowitz, C., Cohen, R. L., & Nettleton, S. (2011). Conceptualising body work in health and social care. *Sociology of Health & Illness*, 33(2), 171–188. 10.1111/j.1467-9566.2010.01323.x 21226736

[cit0032] Vassbø, T. K., Kirkevold, M., Edvardsson, D., Sjögren, K., Lood, Q., & Bergland, Å. (2019). The meaning of working in a person-centred way in nursing homes: A phenomenological-hermeneutical study. *BMC Nursing*, 18(1), 45. 10.1186/s12912-019-0372-9 PMC6790040 31632193

[cit0033] Yeatts, D. E., Seckin, G., Shen, Y., Thompson, M., Auden, D., & Cready, C. M. (2018). Burnout among direct-care workers in nursing homes: Influences of organisational, workplace, interpersonal and personal characteristics. *Journal of Clinical Nursing*, 27(19–20), 3652–3665. 10.1111/jocn.14267 29322572

[cit0034] Yeung, J., Jones, A., Jhangri, G. S., Gibson, W., Hunter, K. F., & Wagg, A. (2019). Toileting disability in older people residing in long-term care or assisted living facilities: A scoping review of the literature. *Journal of Wound, Ostomy and Continence Nursing*, 46(5), 424–433. 10.1097/WON.0000000000000575 31513130

